# Massage in the application of perioperative medicine: research hotspots and trends

**DOI:** 10.3389/fmed.2025.1542450

**Published:** 2025-04-01

**Authors:** Xiaoqin Li, Chuan You, Haige Wei, Huan Li, Yun Liang

**Affiliations:** ^1^Clinical College, Affiliated Hospital of North Sichuan Medical College, Nanchong, Sichuan, China; ^2^Acupuncture and Tuina School, Chengdu University of Traditional Chinese Medicine, Chengdu, Sichuan, China

**Keywords:** massage, perioperative medicine, bibliometric, postoperative period, intraoperative period

## Abstract

**Background:**

Over the past decade, the application of massage in the perioperative period has attracted the interest of researchers worldwide, with a relatively stable volume of literature published annually.

**Objective:**

By conducting a bibliometric analysis of the literature from the past decade, this study aims to identify the research overview, hotspots, and trends of massage in perioperative medicine.

**Methods:**

A search was conducted in the Web of Science Core Collection for literature on the application of massage in perioperative medicine from 2014 to 2024. Articles and reviews were collected with a language restriction to English. Bibliometric and visual analyses were conducted using Co-Occurrence, R Studio, VOSviewer, and Scimago Graphica software.

**Results:**

A total of 434 literature records were retrieved, and after data cleaning (removal of duplicates and incomplete entries), 344 articles were ultimately included. Overall, the number of publications showed a fluctuating upward trend annually. The United States leads in publication volume and has established considerable collaboration with Europe and Brazil, while China ranks second in publication volume but lacks international collaboration. Research primarily focuses on the management of pain and anxiety symptoms, including the improvement of quality of life for patients with somatic diseases and oncological conditions. Cutting-edge topics primarily focus on the efficacy verification of massage techniques and the specific areas targeted, particularly acupoint massage.

**Conclusion:**

This study summarizes the research status, hotspots, and trends of massage in perioperative medicine over the past decade, which may aid researchers in gaining a better understanding of this field. China should strengthen international collaboration to promote the development of this field. The efficacy verification of massage techniques and areas, particularly acupoint massage, will be a focal point for future research.

## 1 Introduction

The perioperative period encompasses the entire process surrounding surgery, commencing with the patient’s decision to undergo surgical treatment and concluding at the completion of postoperative recovery, encompassing multiple stages including preoperative preparation, the surgical procedure itself, and postoperative recovery (1). The patient’s preoperative condition can influence their tolerance of the surgical procedure as well as the likelihood of encountering surgical complications. Moreover, the trauma associated with surgery and anesthesia may precipitate additional complications and sequelae during the postoperative period, consequently prolonging the patient’s recovery prognosis and the temporal costs associated with recuperation (2). Perioperative medicine, a pivotal element of contemporary medical practice, encompasses a spectrum of medical interventions aimed at optimizing preoperative preparation and fostering postoperative recovery, with objectives including the enhancement of surgical success rates, the reduction of hospital stays, and the amelioration of patient prognoses (3). Scientific management of perioperative care must adhere to the tenets of evidence-based medicine, draw upon clinical practice expertise, and be collaboratively developed and executed by a consortium of disciplines to guarantee the holistic and efficacious nature of perioperative medical interventions (4).

Massage, a widely utilized non-pharmacological therapy in physical rehabilitation, encompasses a range of systematic manipulation techniques, including effleurage, petrissage, and tapotement. Among these, acupoint massage, a technique rooted in Traditional Chinese Medicine (TCM), focuses on stimulating specific points on the body to regulate qi and blood flow, thereby promoting healing and relief from various symptoms. Subsequent studies have confirmed that acupoint massage, in particular, provides a multitude of therapeutic benefits during the perioperative period (5).

The primary application of massage in perioperative medicine stems from its role in preventing postoperative venous thrombosis (6). Subsequent studies have confirmed that massage provides a multitude of therapeutic benefits beyond preventing postoperative venous thrombosis during the perioperative period. Massage, when implemented during the perioperative period, can alleviate patient anxiety (7), pain (8), postoperative ileus (9), and other conditions.

Upon conducting a literature search on Web of Science (WoS), a plethora of studies on the application of massage in perioperative medicine were found to have been published over the past decade. Achieving a comprehensive understanding of this field’s current state and development necessitates an investigation into the characteristics, hotspots, and trends of the relevant research. Advancing computer technology has led to the digitization, networking, and intellectualization of information. Bibliometric analysis applies quantitative methods to the study of literature in specific fields, revealing intrinsic connections between pieces of information through literature acquisition and visualization techniques. In recent years, bibliometric analysis has been applied to analyze massage applications for conditions such as lumbar and cervical pain (10, 11). However, the application of massage in perioperative medicine remains unreported.

This study utilizes bibliometric methods to analyze the application of massage in perioperative medicine over the past decade, identifying the hot topics and trends in its use within this field.

## 2 Materials and methods

### 2.1 Search strategy and data retrieval

A comprehensive search was undertaken within the WoS Core Collection to identify literature pertaining to the application of massage, with a specific focus on acupoint massage, within the domain of perioperative medicine spanning the years 2014 to 2024. The search strategy utilized the following terms: (TS = (Post$operat* OR pre$operat* OR intra$operat* OR peri$operat* OR Post$surgical OR pre$surgical) OR TI = (operation OR surgery OR *ectomy)) AND TS = “Tuina” OR “Massage” OR “manual therapy” OR “chiropractic” OR “cheirapsis” OR “rubdown” OR “knead.” The search strategy was tailored to capture studies specifically mentioning or focusing on acupoint massage. The scope of the literature was restricted to articles and reviews, and was limited to English language publications. Subsequently, the identified publications were independently assessed by two authors to ascertain their relevance to the research topic. Any discrepancies were resolved through consultation with a third author. The literature identified was stored in datasets formatted as ‘Plain Text File’ and ‘Full Record and Cited References’. The dataset includes bibliometric data such as the publication’s title, year of publication, authorship, nationality, affiliation, journal of publication, keywords, citation information, type of literature, and abstract. Following this, data cleansing procedures were performed, which included deduplication, data supplementation, and the elimination of duplicate entries. The process of literature screening is depicted in Supplementary Figure 1.

### 2.2 Statistical analysis method

This paper employs Co-Occurrence (COOC) (version 14.5) (12) and R Studio software (version 2024.04.2 + 764, ‘bibliometrix’ package installed) (13) for synonym consolidation, frequency analysis, co-occurrence matrix generation, dissimilarity matrix generation, term-document matrix generation, bimodal matrix generation, cluster diagram creation, research front identification, time zone mapping, and thematic evolution pathway mapping in bibliometric analysis. Additionally, VOSviewer (version 1.6.18) (14) and Scimago Graphica (15) software are utilized for geographical visualization. Initially, a series of manual verifications were performed to standardize the naming conventions for institutions and authors with identical names. This paper delineates the characteristics and knowledge landscape of literature on massage within the perioperative medicine field over the past decade, constructing a collaborative network map encompassing countries, institutions, authors, and journals, and elucidating the contributions and collaborative efforts of the papers. Furthermore, the analysis of keyword co-occurrence, clustering, timelines, and thematic evolution-accumulative time zone diagrams has facilitated the identification of research hotspots and trends.

## 3 Results

### 3.1 Annual numbers of publications

**Figure 1:**
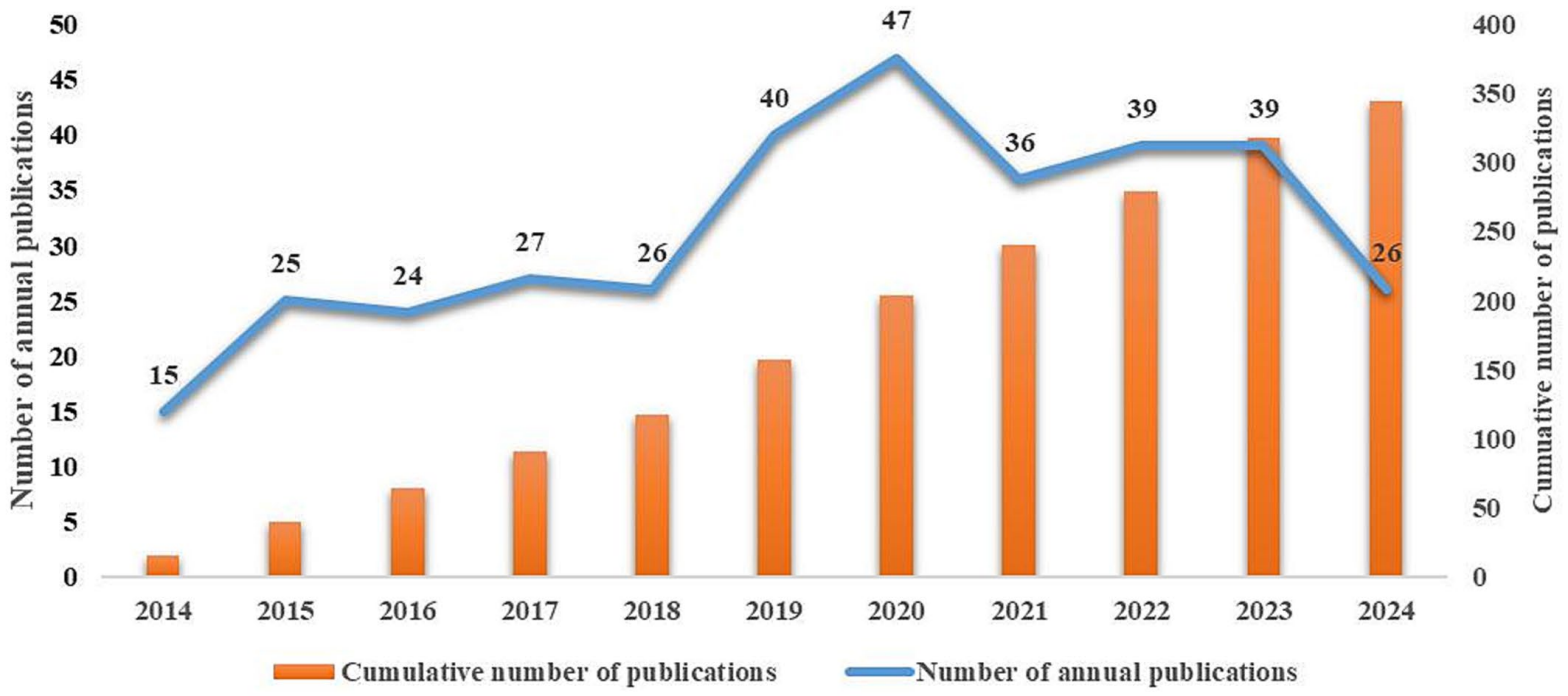
The annual and cumulative number of publications from 2014 to 2024.

A search of the WoS Core Collection yielded a total of 434 records from January 2014 to August 2024, comprising 325 articles and 88 reviews. Following the removal of duplicate titles (*n* = 1) and data cleaning (*n* = 68), a total of 344 publications were included in the analysis (see RAW Data Files). The publication selection flowchart for this study is detailed in Supplementary Figure 1. Figure 1 displays the annual publication count related to massage in perioperative medicine over the past decade. Throughout the past decade, the number of articles published in this field fluctuated, increasing from 2014 to 2020, then declining after 2020. Overall, however, the annual publication count showed a fluctuating upward trend.

### 3.2 Distribution of countries/regions

**Figure 2:**
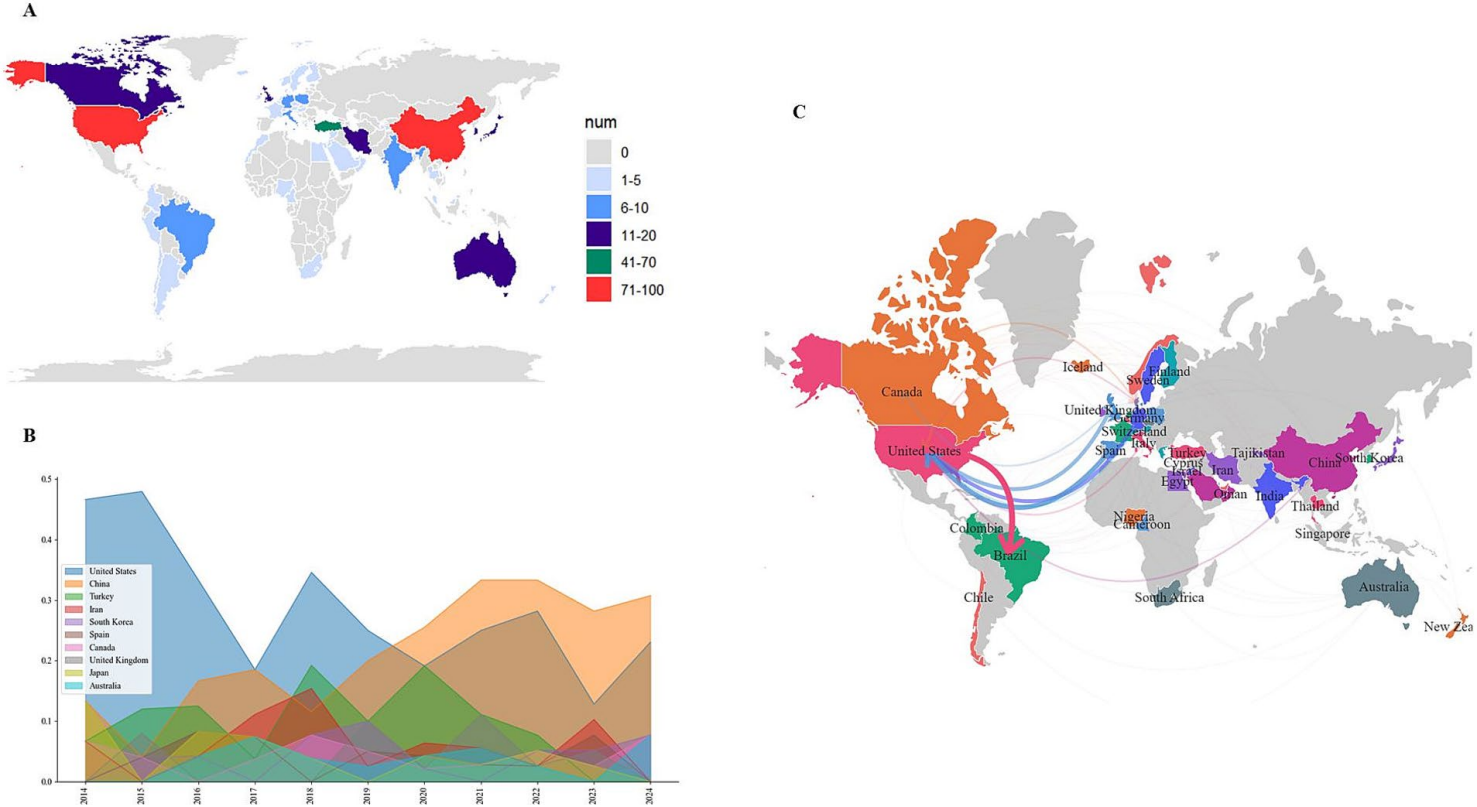
**(A)** Number of publications by country/region; **(B)** growth rate of annual publications for the top 10 countries/regions by publication count; **(C)** collaboration network map among countries.

From 2014 to 2024, scholars from 55 countries and regions contributed to a corpus of 344 publications, either independently or through collaborative efforts. Figure 2A shows the distribution of these countries and regions based on their publication output over the past 10 years, highlighting the United States with a leading 91 publications, followed by China with 79 and Turkey with 33. Figure 2B presents the year-on-year growth trend of publication volumes for the leading ten countries and regions in the field of massage therapy within perioperative medicine. The United States showed the most robust growth between 2014 and 2019. Subsequently, China surpassed all others in publication growth rate after 2019. Since 2020, Spain has also exhibited a commendable growth trajectory in its publication counts. Figure 2C shows a network map of collaborative ties among these countries. The United States emerges as a pivotal actor in this research field. Many European countries play a secondary role in collaborations with the United States, in contrast to Brazil, which takes a leading role in its partnerships with the United States. Notably, China, ranking second in publication volume, exhibits only a tenuous collaborative relationship with the United States and appears to lack an extensive collaborative framework with other countries.

### 3.3 Analysis of keywords

Keywords distill and encapsulate the research content and thematic elements of an article. Analysis of the 344 publications yielded 1,084 unique keywords.

**Figure 3:**
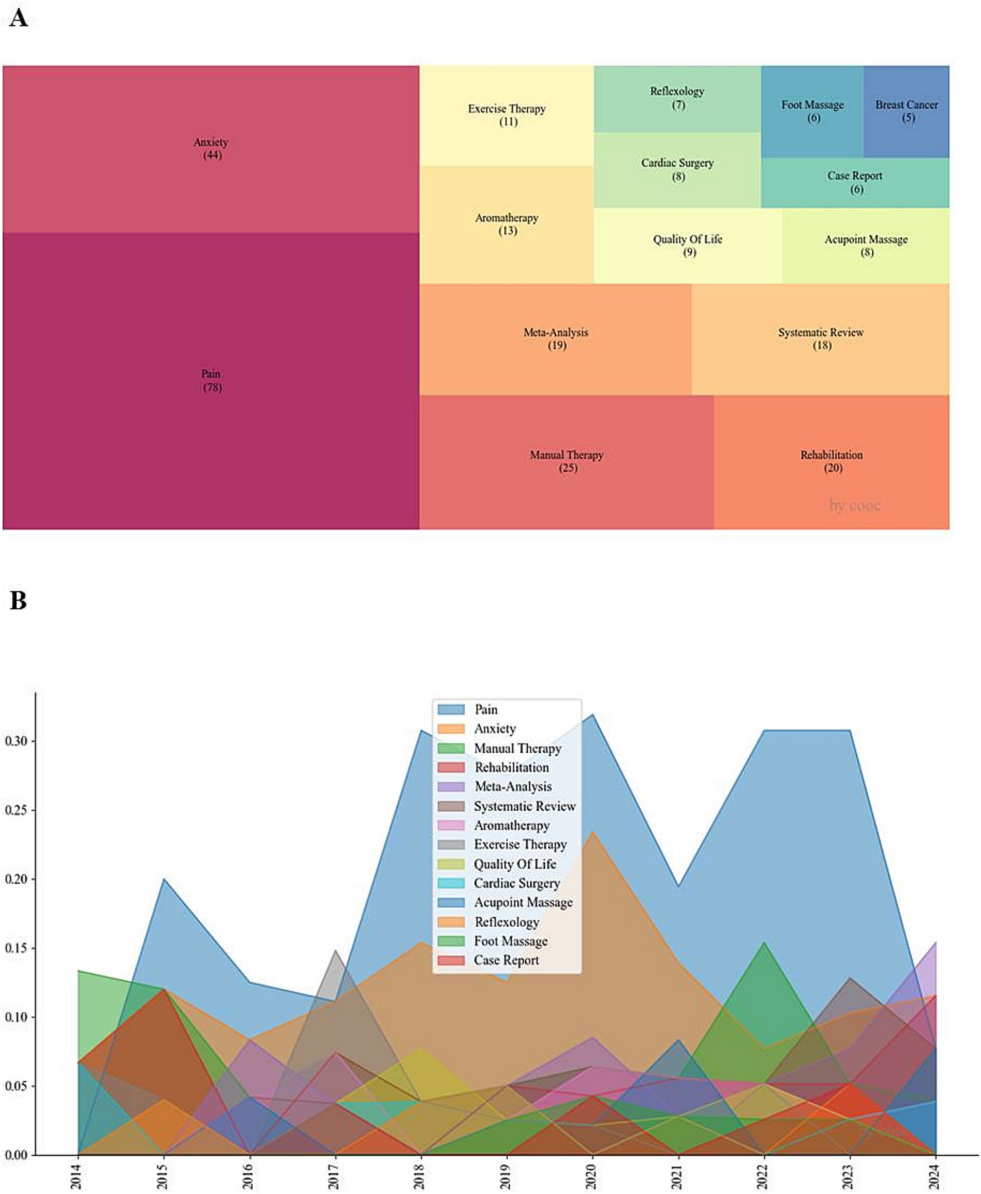
**(A)** Tree map of the top 15 keywords by frequency; **(B)** annual attention to the top 14 keywords by frequency.

Figure 3A displays a dendrogram of the 15 most frequent keywords, excluding the search terms ‘manual therapy,’ ‘surgical,’ and ‘postoperative.’ The prominence of ‘Pain’ (*n* = 78), ‘Anxiety’ (*n* = 44), ‘Rehabilitation’ (*n* = 20), and ‘Quality of life’ (*n* = 9) suggests that the primary focus of massage therapy is to alleviate pain and anxiety symptoms and enhance recovery and quality of life in the perioperative period. The terms ‘Meta-Analysis’ (*n* = 19), ‘Systematic Review’ (*n* = 18), and ‘Case Report’ (*n* = 6) correspond to the categories of research studies. Keywords such as ‘Aromatherapy’ (*n* = 13), ‘Exercise Therapy’ (*n* = 11), ‘Acupoint Massage’ (*n* = 8), ‘Foot Massage’ (*n* = 6), and ‘Manual Therapy’ (*n* = 25) indicate specific massage therapy modalities. The keywords ‘Cardiac Surgery’ (*n* = 8) and ‘Breast Cancer’ (*n* = 5) specify the disease types to which massage therapy is applied.

Figure 3B depicts the annual research interest in the 14 most frequent keywords. Research on ‘Pain’ has consistently garnered high interest. Interest in ‘Anxiety’ declined in 2022, followed by a resurgence. The annual interest in the remaining keywords has remained stable; however, the annual interest in ‘Meta-Analysis,’ ‘Case Report,’ and ‘Acupoint Massage’ has shown an increasing trend.

**Figure 4:**
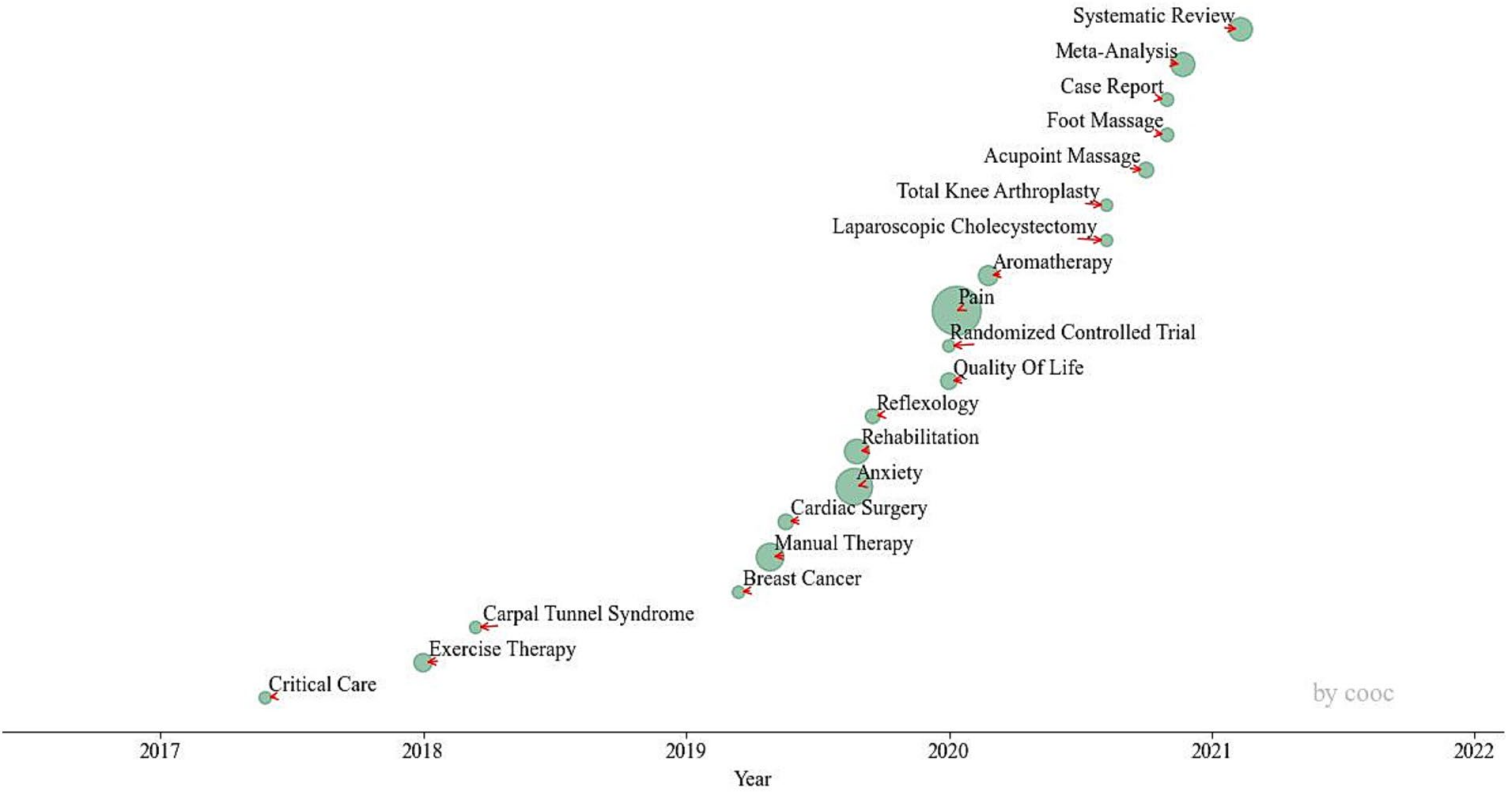
Weighted time zone map based on keyword theme evolution.

Figure 4 presents a weighted time zone map delineating the thematic evolution of keywords, with a minimum frequency threshold set at 5 occurrences. The horizontal axis shows the average year each keyword appears, while the diameters of the green circles correspond to the cumulative frequency of keyword appearances. This method multiplies the frequencies of a keyword’s appearances across various years and then sums these values. It culminates in calculating the average year of appearance for each keyword, reflecting the trajectory of research topic evolution. From 2017 to 2021, research on massage application during the perioperative period focused predominantly on applicable diseases, including “Critical Care,” “Carpal Tunnel Syndrome,” “Breast Cancer,” “Cardiac Surgery,” “Laparoscopic Cholecystectomy,” and “Total Knee Arthroplasty.” Post-2021, research in the field of massage during the perioperative period has shifted toward “Meta-Analysis” and “Systematic Review,” which conduct retrospective and comprehensive analyses of prior studies.

**Figure 5:**
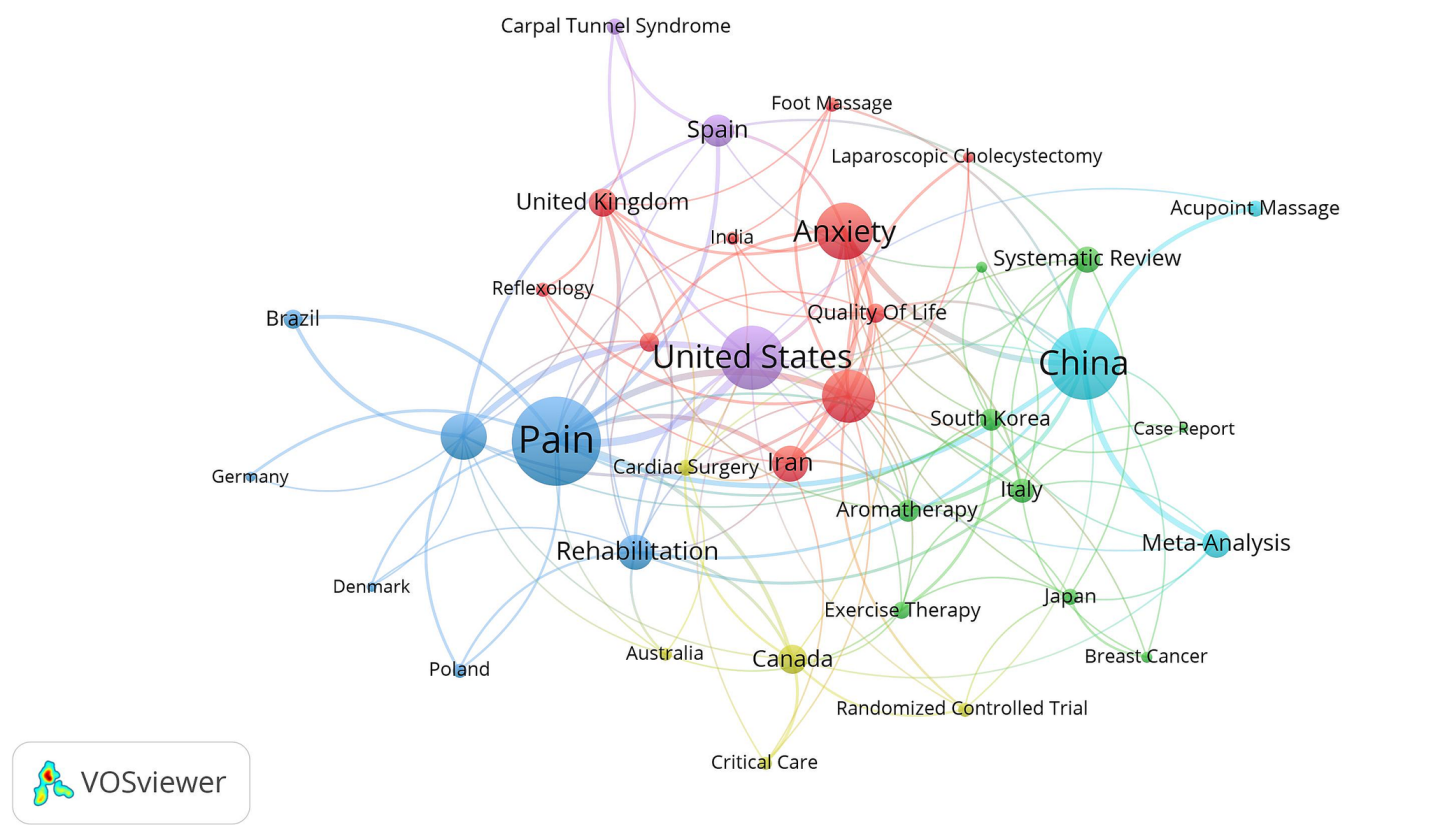
Bipartite network graph of countries and keywords.

Figure 5 presents a bipartite network graph illustrating the collaborative relationships between major countries and their research domains. The United States and Spain, leading in publication volumes, are mainly linked to research on Carpal Tunnel Syndrome. China, ranking second in publication volume, shows no collaborative ties with other countries and is focused on Acupoint Massage and Meta-Analysis. The keyword ‘Pain,’ the most frequently occurring, is mainly researched by Poland, Brazil, Denmark, and Germany. Concurrently, the United Kingdom, Iran, India, and Turkey focus their research efforts on Anxiety.

**Figure 6:**
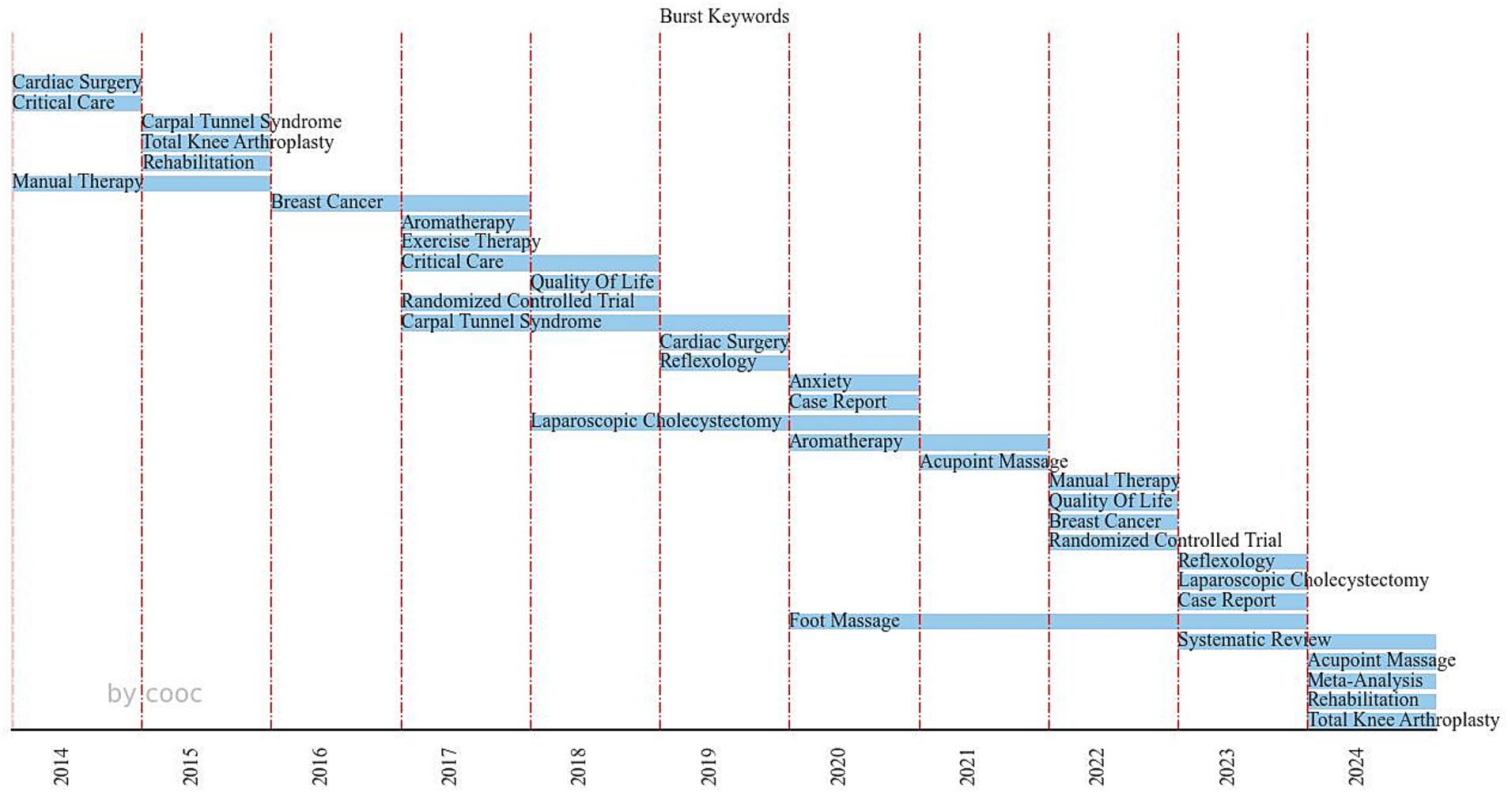
Analysis of burst keywords (with a frequency threshold of ≥5).

Figure 6 provides an analysis of Burst Keywords with a frequency greater than 5. Burst Keyword analysis quantifies research hotspots in a field over different periods and is commonly used to predict the development trends in that field qualitatively. Burst Keyword analysis identifies keywords with significant frequency changes over a short period and measures the start and end points of the keyword’s appearance in the Burst Keyword map. This duration is referred to as the keyword’s burst duration, and the observed keyword is termed a Burst Keyword. The closer the appearance and end points of a Burst Keyword are to the present, the more likely the keyword represents a future research trend. Keywords with a burst duration exceeding 2 years include “Manual Therapy,” “Breast Cancer,” “Critical Care,” “Randomized Controlled Trial,” “Carpal Tunnel Syndrome,” “Laparoscopic Cholecystectomy,” and “Foot Massage.” Figure 6 indicates that research hotspots for 2024 and beyond are “Systematic Review,” “Acupoint Massage,” “Meta-Analysis,” “Rehabilitation,” and “Total Knee Arthroplasty.”

## 4 Discussion

Massage, an ancient therapeutic practice originating from the human instinct to alleviate pain through touch, has been widely adopted across the globe, including regions such as China, Europe, the Americas, and Africa. This study undertook a bibliometric analysis of the application of massage in perioperative medicine over the past decade, utilizing the WoS Core Collection to summarize general information, research hotspots, and trends in this field. A total of 344 publications were included in this study. From 2014 to 2024, the number of annual publications in this field showed a fluctuating increase until 2020, after which there has been a decline, suggesting that there remains significant potential for heightened focus on the application of massage in the perioperative period.

### 4.1 General information

Figure 2A illustrates the geographical distribution of the 344 publications, originating from a diverse array of countries spanning the Americas (including the United States, Canada, and Brazil), Europe (encompassing the United Kingdom, Spain, Germany, Turkey, and Italy, among others), Asia (with contributions from China, South Korea, Japan, and India), and Oceania (comprising Australia and New Zealand). The United States holds the foremost position in terms of publication numbers and sustained a prominent dominance up until 2019, as evidenced by Figure 2B. It has forged strong collaborative ties with numerous European nations and Brazil, thereby highlighting the pivotal role that the United States occupies within this research domain. Despite ranking second in publication numbers and emerging as the nation with the highest growth rate in publications since 2019, China has yet to establish substantial collaborative networks with other countries, with the exception of a tenuous partnership with the United States. This underscores the necessity for China to enhance its engagement with the global research community.

### 4.2 Research hotspots of massage in perioperative medicine

Keywords serve as concise encapsulations of an article’s research content and thematic information. By conducting a thorough keyword analysis this study has illuminated the key research hotspots within the field of perioperative massage therapy thereby clarifying the overarching research direction.

Figure 5, which presents a bipartite network graph analysis of countries and keywords, unveils that both the “United States” and “Spain” harbor a shared research emphasis on conditions arising from peripheral nerve compression, exemplified by “Carpal Tunnel Syndrome” (16, 17). “South Korea,” “Italy,” and “Japan” exhibit a collective research emphasis on the adjunctive treatment of joint diseases, exemplified by “Total Knee Arthroplasty” (18, 19), as well as oncological patients, particularly those with “Breast Cancer” (20), employing specific massage modalities such as “Aromatherapy” (21), and “Exercise Therapy” (22), which are frequently substantiated through comparative studies. “Systematic Review” and “Case Report” constitute frequently utilized research methodologies. “Poland,” “Brazil,” “Denmark,” and “Germany” direct their research efforts toward the management of “Pain” during the perioperative period (23). “China” exhibits a particular focus on “Acupoint Massage” (24), demonstrating a predilection for application-oriented research that integrates acupoint stimulation with massage techniques. Furthermore, China consolidates the application of massage during the perioperative period by employing “Meta-Analysis.” Meanwhile, “United Kingdom,” “Iran,” “India,” and “Turkey” prioritize the alleviation of “Anxiety” during the perioperative period (25, 26).

By integrating the annual attention to keywords depicted in Figure 3B with the topic evolution illustrated by the weighted time zone map in Figure 4, research conducted between 2017 and 2021 on the application of massage in the perioperative period centered on validating treatment outcomes for a range of diseases, including “Critical Care,” “Carpal Tunnel Syndrome,” “Breast Cancer,” among others. Following 2021, there has been a notable dearth of innovative research, with the exception of efficacy validation studies on “Acupoint Massage,” which predominantly comprise articles of the “Meta-Analysis” and “Review” types.

### 4.3 Research trend of massage in perioperative medicine

“Burst Keywords” refer to terms that exhibit a surge in frequency over a defined timeframe, facilitating the examination of trends in field development. The analysis of Figure 6 reveals that the research on perioperative massage application is quite dispersed, with a limited number of sustained research hotspots spanning beyond a two-year period. It is anticipated that “Acupoint Massage,” “Total Knee Arthroplasty,” “Meta-Analysis,” and “Rehabilitation” will emerge as prominent research hotspots in the future. Taking into account the earlier bipartite network analysis of countries and keywords, “Acupoint Massage” has been pinpointed as the focal points of research in China. Moreover, given that China has exhibited the highest growth rate in publications within this field since 2019, it is foreseen that China will assume a pivotal role in future research endeavors. Post-operative edema and pain resulting from Total Knee Arthroplasty have the potential to impede the recovery of muscle function. A comprehensive search of the literature database uncovers considerable research on Total Knee Arthroplasty conducted in countries such as Japan, South Korea, and Italy, covering various aspects including surgery, recovery, nursing, and influencing factors. Massage, serving as a complementary therapy, is poised to remain a prominent area of investigation for its techniques and applications, in conjunction with foundational studies.

Research on ‘Pain’ has consistently garnered high interest, with acupoint massage emerging as a promising non-pharmacological intervention. Studies have demonstrated that acupoint massage can effectively reduce postoperative pain, likely through mechanisms involving the regulation of pain-related neurotransmitters and the modulation of the central nervous system’s pain perception pathways. The annual interest in ‘Acupoint Massage’ has shown an increasing trend, reflecting the growing recognition of its potential benefits in the perioperative setting. Furthermore, acupoint massage has also been shown to alleviate anxiety, improve sleep quality, and enhance overall recovery outcomes, suggesting its multifaceted therapeutic effects. Future research should focus on elucidating the precise mechanisms underlying these effects and optimizing the application of acupoint massage in various surgical contexts.

## 5 Conclusion

This study summarizes the research status, hotspots, and trends of massage in perioperative medicine over the past decade. Our findings highlight the growing interest in and potential benefits of massage for managing pain, anxiety, and other symptoms during the perioperative period. The efficacy verification of acupoint massage techniques, as well as the elucidation of their underlying mechanisms, will be focal points for future research. Additionally, the optimization of acupoint massage protocols for different surgical contexts represents an important area of exploration.

However, the study has several limitations, including the inclusion of only English-language studies and the exclusion of gray literature and conference proceedings. Future research should aim to address these limitations and provide a more comprehensive assessment of the field. Additionally, greater international collaboration, especially among Chinese researchers, is needed to advance the development of massage in perioperative medicine.

## 6 Future research directions

Future studies should focus on the efficacy and mechanisms of specific massage techniques, such as acupoint massage. Moreover, a broader inclusion of literature, including non-English studies and gray literature, is warranted to provide a more comprehensive understanding of the field. Additionally, clinical trials and comparative studies are needed to validate the findings of current research and guide the implementation of massage in perioperative care.

## Data Availability

The original contributions presented in the study are included in the article/Supplementary material, further inquiries can be directed to the corresponding author.
