# Supplementary material for: Massage in the application of perioperative medicine: research hotspots and trends
Source: Front Med (Lausanne). 2025 Apr 1;12:1542450. doi: 10.3389/fmed.2025.1542450 (PMC11998281; doi:10.3389/fmed.2025.1542450)
Supplement: Supplementary file 1 [file Supplementary_file_1.docx]

Massage in the Application of Perioperative Medicine: Research Hotspots and Trends

**Xiaoqin Li ^1^, Chuan You ^1^, Haige Wei^1^ ,Huan Li^1^ , Yun Liang^1,2,^ ***

^1^Clinical College, Affiliated Hospital of North Sichuan Medical College, Nanchong, Sichuan, 637000, P. R. China

^2^Acupuncture and Tuina School, Chengdu University of Traditional Chinese Medicine, Chengdu, Sichuan, 610075, P.R. China

*** Correspondence:**Yun Liang

Email: liangyun@nsmc.edu.cn


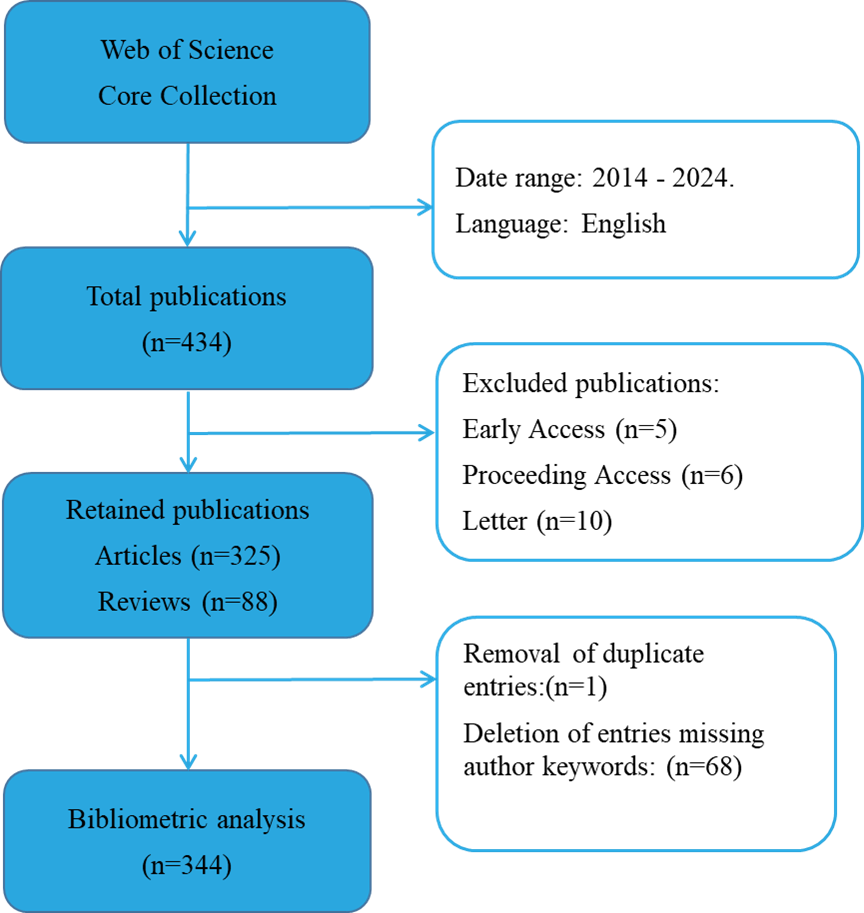


**Supplementary Figure 1.** The publication selection flowchart for this study.
